# Endoscopic ear surgery in Canada: a cross-sectional study

**DOI:** 10.1186/s40463-016-0117-7

**Published:** 2016-01-19

**Authors:** Michael Yong, Tamara Mijovic, Jane Lea

**Affiliations:** University of British Columbia, Division of Otolaryngology – Head and Neck Surgery, 4th Floor, 2775 Laurel Street, Vancouver General Hospital, Vancouver, BC V5Z 1 M9 Canada; McGill University, Department of Otolaryngology – Head and Neck Surgery, Royal Victoria Hospital - D05.5712, 1001 Décarie Boul, Montreal, H4A 3 J1 Canada; University of British Columbia, Division of Otolaryngology – Head and Neck Surgery, ENT Clinic, 1081 Burrard Street, St. Paul’s Hospital, Vancouver, BC V6Z 1Y6 Canada

**Keywords:** Endoscopic ear surgery, Middle ear surgery, Otology, Survey, Canada

## Abstract

**Background:**

Endoscopic ear surgery is an emerging technique with recent literature highlighting advantages over the traditional microscopic approach. This study aims to characterize the current status of endoscopic ear surgery in Canada and better understand the beliefs and concerns of the otolaryngology – head & neck surgery community regarding this technique.

**Methods:**

A cross-sectional survey study of Canadian otolaryngologists was performed. Members of the Canadian Society of Otolaryngology were contacted though an online survey carried out in 2015.

**Results:**

The majority of participants in this study (70 %) used an endoscope in their practice, with a large proportion utilizing the endoscope for cholesteatoma or tympanoplasty surgery. To date, 38 Canadian otolaryngologists (70 % of respondents) have used an endoscope for at least 1 surgical case, but only 6 (11 %) have performed more than 50 endoscopic cases. Of the otolaryngologists who use endoscopes regularly, the majority still use the microscope as their primary instrument and use the endoscope only as an adjunct during surgery. However, the general attitude surrounding endoscopes is positive; 81 % believe that endoscopes have a role to play in the future of ear surgery and 53 % indicated they were likely to use endoscopes in their future practice. Participants who were earlier in their practice or who had more exposure to endoscopic techniques in their career were more likely to have a positive stance towards endoscopic ear surgery (*p* < 0.05, *p* < 0.01, respectively). The main concern regarding endoscopic ear surgery was the technical challenge of one-handed surgery, while the primary perceived advantage was the reduced rates of residual or recurrent disease.

**Conclusions:**

Endoscopic ear surgery is a new technique that is gaining momentum in Canada and there is enthusiasm for its incorporation into future practice. Further investment in training courses and guidance for those looking to start or advance the use of endoscopes in their practice will be vital in the years to come.

**Electronic supplementary material:**

The online version of this article (doi:10.1186/s40463-016-0117-7) contains supplementary material, which is available to authorized users.

## Background

The use of endoscopes in ear surgery began approximately forty years ago; however, it is only recently that enthusiasm for this technique has grown. Acceptance of endoscopic ear surgery techniques has likewise grown [1], albeit slowly and with initial great resistance. Over the past decade, numerous studies have been published on the overall efficacy of endoscopic ear surgery as compared to the traditional microscopic approach, thus promoting wider usage of the endoscope [2–6]. The endoscope has been supported as a tool for improving the visual exposure of hidden structures and deep recesses, obtaining a wider angle of view, and achieving a minimally-invasive operation with greater healthy tissue preservation [4–6]. The ability to view blind spots during surgeries for diseases such as cholesteatoma has also been shown to decrease residual disease and recurrence rates when compared to surgeries which used the microscope alone [6, 7].

While some authors are optimistic that endoscopes will become increasingly utilized and important in otologic surgery due to the cumulative advances in technique and quality of equipment [4–6], there are still some concerns over safety and efficiency that contribute to the reluctance of some ear surgeons to adopt usage of this technique. Careful control of hemorrhage, anti-fogging methods, reducing potential endoscope-associated thermal injury, and compensation for the loss of depth perception are challenges that need addressing when maximizing the safety of the procedure [5, 8, 9]. In addition, the cost of endoscopic equipment and the need for specialized training and experience is a hurdle that can further deter surgeons who already practice exclusively with the microscope from embracing this new technique [5, 8].

At the present time, there are no studies that characterize the usage patterns of endoscopes among those who perform ear surgery in Canada. Given the improvements in technology and changes made to the endoscopic technique over the past four decades, an assessment of the current attitudes towards endoscopic ear surgery will provide some valuable insight on the role this approach currently plays and may play in the future. The objective of this study was to provide an analysis of the current usage of endoscopic ear surgery techniques among Canadian otolaryngologists, as well as obtain a better understanding of the attitudes and learning experiences surrounding endoscopic ear surgery in Canada.

## Methods

Following approval by the UBC Behavioural Research Ethics Board (ID H14-03499), members of the Canadian Society of Otolaryngology were contacted by email and invited to participate in an on-line survey. Subject invitation and recruitment were facilitated using the Canadian Society of Otolaryngology’s e-mail listserv and took place during a 6-week period from March 2015 to April 2015. Consent was obtained from each study participant to use the anonymous study data collected for the purposes of publication.

This cross-sectional study involved an online survey questionnaire administered through FluidSurveys (Ottawa, ON, Canada). It was composed of eleven main questions (Additional file 1) aimed at characterizing the subjects’ surgical experience, use of endoscopes in ear surgery, and perceived advantages and concerns with endoscopic ear surgery techniques. This survey was not pre-validated because no similar characterizations of endoscope usage have been previously conducted.

### Statistical analysis

Descriptive statistics were used to characterize the current use of endoscopes and identify the concerns and attitudes held by otolaryngologists regarding the use of endoscopes in ear surgery.

Study participants were divided into categories based on the number of years in practice and the number of endoscopic ear cases performed. These two categorical sub-groups were then used as factors against which the responses to various continuous and categorical variable survey questions were analyzed. In particular, three main questions regarding the likelihood to use endoscopes in the future, overall learning experience with endoscopes, and belief in a role for endoscopes in ear surgery in the future were chosen for statistical analysis. Furthermore, three additional questions regarding concerns, advantages, and ease of use were analyzed for descriptive purposes.

Odds ratio calculations comparing various pre-determined sub-groups were conducted for three key questions which were felt to best represent the overall attitude towards using endoscopes in ear surgery (Questions 7, 10, and 11). In addition, cross-tabulations (Pearson’s chi-square test and Fishers exact test for small sample sizes) were conducted for the categorical data in Questions 7 and 10 and one-way ANOVA analysis was conducted for the continuous variable data in Question 11. Rigorous statistical analysis excluded data from resident physicians due to lack of adequate sample size. These data were still included in the reported percentages in the descriptive statistics. All data was analyzed using Excel 2013 (Version 15.0, Microsoft®).

## Results

### Study participants

**Fig. 1:**
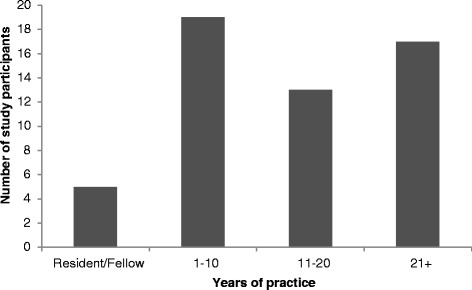
Distribution of study participants by number of years in practice

The survey was sent to 703 individuals; 484 active, 50 emeritus, and 169 resident members of the Canadian Society of Otolaryngology. At the conclusion of the 6-week study period, 80 surveys were completed. Of these 80 responses, 16 were incomplete with no usable data and discarded. Of the remaining 64 responses, 10 were from otolaryngologists who did not perform ear surgery and were therefore excluded, leaving 54 responses for analysis. Of the 54 study participants, 16 (30 %) were otologists, 21 (39 %) were general otolaryngologists, 12 (22 %) were paediatric otolaryngologists, and 5 (8 %) were trainees (residents and fellows). Figure 1 describes the distribution of the number of years subjects have been in practice.

### Use of endoscopes in ear surgery practice

**Fig. 2:**
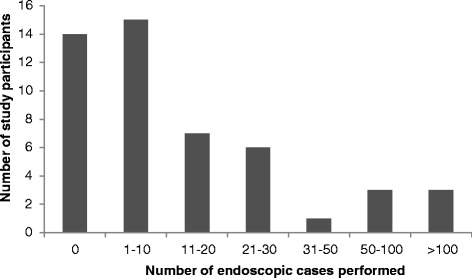
Distribution of study participants by number of endoscopic ear cases performed

**Fig. 3:**
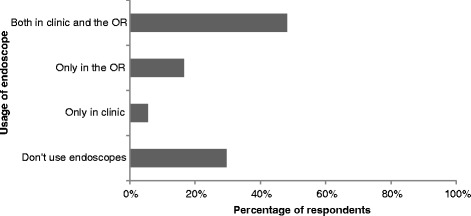
Usage of endoscopes in otology practice

Among the respondents who perform ear surgery, 70 % indicated that they use endoscopes in their practice. Figure 2 describes the number of endoscopic cases that respondents have performed. Based on our survey, there are currently 38 surgeons in Canada who have performed at least one endoscopic ear case, but only 6 surgeons have completed more than 50 cases. Of the surgeons who indicated that they use an endoscope, 68 % used the endoscope in the clinic and in the operating room, while smaller numbers of surgeons used the endoscope only in clinic (8 %) or only in the operating room (24 %) (Fig. 3).

**Fig. 4:**
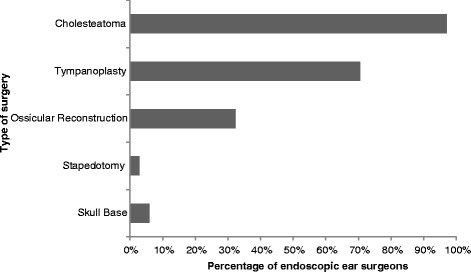
Use of the endoscope in specific types of ear surgery, expressed as percentage of surgeons who actively use an endoscope in the operating room

**Fig. 5:**
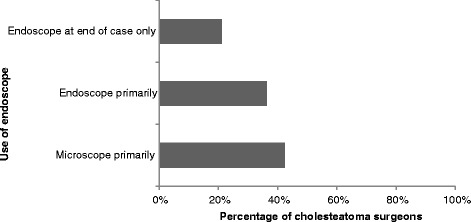
Use of the endoscope during cholesteatoma surgery, expressed as percentage of surgeons who perform cholesteatoma surgery

Cholesteatoma (97 %) and tympanoplasty (71 %) were the two most common applications for the endoscope among respondents (Fig. 4). Ossicular reconstruction was a more infrequent application, while skull base and stapedotomy were very uncommon uses. Specifically for cholesteatoma surgery, 42 % of surgeons still primarily use the microscope with the endoscope as an adjunct, 36 % mainly use the endoscope, and 21 % only use the endoscope to check for residual disease at the end of the case (Fig. 5).

### Attitude towards endoscopes in ear surgery

The majority of participants (81 %) recognize a role for the endoscope in ear surgery. The recognition of a role for endoscopes was seen across sub-groups including those who do not perform endoscopic surgery (57 %) and those who were well into their practice (65 %). There was no statistically significant difference among the sub-groups.

Overall, participants had a positive stance on endoscopic ear surgery with over 50 % indicating that they were likely to use endoscopes for ear surgery in their future practice (Table 1). There was a significant difference in the likelihood of using endoscopes in the future based on number of years in practice (*p* < 0.05), as well as based on number of endoscopic cases performed to date (*p* < 0.01). Study participants indicated that they were more inclined to use endoscopes for ear surgery in their future practice if they were earlier in their practice with 11 to 20 years of experience (OR 2.33, 95 % CI 0.05–11.81) and significantly more inclined with only 1 to 10 years of experience (OR 18.67, 95 % CI 1.88–185.41, *p* < 0.01) when compared to those with 21 years or more of experience. Participants also responded that they would be more likely to use endoscopes in the future as their endoscopic ear surgery experience increased; when compared to those who had not performed any endoscopic surgery, participants were more likely to use an endoscope in the future if they had done between 1 and 20 endoscopic cases (OR 2.92, 95 % CI 0.55–15.56) and significantly more likely to use endoscopes in the future if they had done more than 20 endoscopic cases (OR 31.29, 95 % CI 1.72–897.14, *p* < 0.05).

**Table 1:** ᅟ

	Mean Rating (0 – Strongly Disagree, 5 – Strongly Agree)	Odds Ratio	95 % Confidence Interval	One-Way ANOVA
Overall (*n* = 50)	3.9	-	-	-
Years in Practice				*p* = 0.014
21+ (*n* = 13)	3.2	1	-	
11-20 (*n* = 12)	4.1	2.33	0.05–11.81	
1–10 (*n* = 17)	4.6	18.67	1.88–185.41*	
Trainee (*n* = 5)	3.8	N/A	-	
Number of Endoscopic Ear Cases				*p* = 0.0030
0 (*n* = 8)	3	1		
1–20 (*n* = 22)	3.9	2.92	0.55–15.56	
21+ (*n* = 12)	4.8	31.29	1.72–897.14*	

Likelihood of using endoscopes in the future, with responses separated by years of practice and by number of endoscopic ear cases performed

*p <0.05

**Fig. 6:**
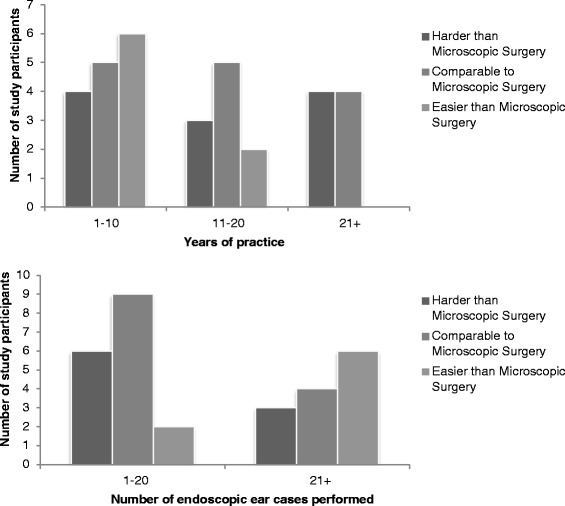
Overall learning experience with endoscopic ear surgery when compared to microscopic surgery, plotted against number of years in practice and against number of endoscopic ear cases performed

Participants also appeared to be more likely to find endoscopic ear surgery easier than microscopic surgery if they were earlier in their practice and if they had done more endoscopic ear cases to date, but no significant difference was found between these sub-groups (Fig. 6).

### Concerns and challenges surrounding endoscopic ear surgery

Single-handed surgery was the main prevailing concern regarding endoscopic ear surgery (44 %), followed by efficiency/operative time (32 %), technical difficulty (25 %), cost (24 %), and managing bleeding (24 %). No concerns over endoscopic surgery were expressed by 36 % of participants.

### Advantages of endoscopic ear surgery

Reduced recurrence and residual disease rate was the most frequent perceived advantage (59 %), followed by ease of teaching trainees (36 %), faster patient recovery (31 %), ease of use (25 %), and less post-operative pain (25 %). No advantage to endoscopic ear surgery over microscopic ear surgery was expressed by 17 % of participants.

## Discussion

The field of ear surgery has seen rapid technological advancement that has greatly impacted the field of otology, first with the invention of the operating microscope, and more recently with the emergence of minimally-invasive endoscopic techniques. Numerous advantages of the endoscope as compared to the microscope have been described and it has become clear that despite some of the disadvantages of the endoscopic approach, such as technical skill necessary and increased training requirements, many ear surgeons recommend a move towards minimally-invasive endoscopic ear surgery techniques.

Although the response rate of our study limits our ability to accurately characterize the use of endoscopic ear surgery in Canada, this survey shows an interest among otolaryngologists in Canada regarding endoscopic ear surgery techniques with several surgeons already adopting and implementing the technique in the operative setting. In addition, there appears to be prevailing optimism regarding the future role of the endoscope in otologic surgery, even among those not currently using endoscopes. Based on our survey, the most common application for the endoscope in the operating room is among cholesteatoma and tympanoplasty procedures. This finding is consistent with previous literature which describes reduced cholesteatoma recurrence rates when using an endoscope and the advantages of minimally-invasive surgery in surgeries such as tympanoplasty [4, 6, 10,11]. However, among our respondents it seems that the microscope is still the instrument of choice in Canada for these procedures due to the amount of study participants indicating that they use the endoscope only as a adjunct or to check for residual disease at the end of a case. While the endoscope is being used for other purposes such as ossicular reconstruction, skull base surgery, and stapedotomy, it appears that the endoscopic approach in these procedures is not common practice among the subgroup of Canadian otolaryngologists who responded to the survey.

Challenges surrounding the technical skill required continue to deter some surgeons from using endoscopes [8]. The main concerns regarding the use of endoscopes in ear surgery based on this study were single-handed surgery, efficiency, cost, and technical difficulty; similar concerns have been raised in previously published literature, especially the challenge of one-handed surgery and the initial technical difficulty of implementing and using endoscopic equipment for the surgeon and operative team [6, 8, 11]. Nonetheless, most surgeons indicated that they were likely to use endoscopes for ear surgery in their future practice. In particular, those surgeons who were earlier in their practice and had performed more endoscopic ear cases to date were the most enthusiastic. This supports the concept that surgeons who have a younger practice and a baseline skill level with endoscopes appear to be more likely to invest resources in acquiring endoscopic equipment that may put them in a better position to overcome some of the concerns that deter usage of the endoscope in ear surgery. In the authors' experience, the cost of implementing endoscopic ear surgery is often quite minimal as most centres are often already well-equipped with endoscopic sinus surgery equipment and already have standard otologic operative instruments which, when combined, are more than adequate to get started with the technique.

There is a challenging learning curve when transitioning from the microscope to the endoscope in ear surgery. As expected, this study shows a trend, albeit not statistically significant, that implies that junior staff and those with more endoscopic ear experiences find endoscopic techniques easier than microscopic approaches. This concept has been supported by authors who have described their own endoscopic learning experience and who have also provided guidance to surgeons who are seeking to implement endoscopic techniques into their practice for the first time [8, 11, 12]. Further investment in endoscopic training programs may allow more surgeons to overcome the hurdles that currently preclude them from incorporating endoscopes into their ear surgery practice.

Once some of the initial technical and learning difficulties of endoscopic ear surgery can be overcome, many authors advocate that the benefits of using the endoscope are multifaceted [2, 6, 8, 11]. The proposed benefits of endoscopic surgery based on current literature align well with the views of Canadian otolaryngologists, particularly with respect to the reduced rate of residual or recurrent cholesteatoma and the ease of obtaining better surgical visualization [6, 7]. Other advantages such as reduced post-operative pain and faster patient recovery are also in agreement with literature articles studying these outcomes in ear surgery patients [10, 12, 13].

There was considerable agreement among respondents that endoscopes have a role to play in the future of ear surgery. The fact that there was no significant difference in these results based on either number of years in practice or experience with the endoscope suggests that there is a general acceptance and support for the use of endoscopes in ear surgery among the subgroup of Canadian otolaryngologists participating in the survey. This finding supports previous literature which promotes the use of endoscopes in the field of ear surgery [6–8, 10–11, 14], and, at the very least, indicates that investing further resources into teaching and promoting the use of endoscopes will likely be met with enthusiasm.

This study has a number of limitations. Survey data was collected in a non-randomized manner and was entirely dependent on the voluntary response rate among otolaryngologists who were subscribed to the Canadian Society of Otolaryngology listserv. The survey was sent to 703 Canadian Society of Otolaryngology members and while 64 responded, only 54 actually performed ear surgery in their current practice. Although the response rate was low at 9 %, one must take into account the current subspecialized nature of otolaryngology in Canada within both community and academic environments. Many Canadian otolaryngologists with interests in subspecialty fields such as rhinology/sinus, head and neck, and facial plastics likely ignored the email given that ear surgery is not within their scope of current practice. The response rate, although quite low, is therefore still difficult to fully interpret. Among the active members of the Canadian Society of Otolaryngology, 36 are fellowship trained in otology and 18 have an interest in otology without formal fellowship training. It may not be coincidence that this sum equals 54, which is the exact number of survey responses analyzed for this study. This survey may therefore be biased towards surgeons with a subspecialty interest in otology and likely a practice with higher volumes of more complex otologic surgery. Accordingly, the results need to be interpreted within that context and not extrapolated to the wider otolaryngology community within Canada. The small response rate also meant that in cases where sub-group analysis was necessary, some sample sizes were too small to conduct rigorous statistical analyses. Finally, the amount of trainees completing the survey was too small to be included in rigorous statistical analysis calculations and, thus, was only included in the graphical statistics.

## Conclusion

This is the first study aimed toward comprehensively characterizing the current state of endoscopic ear surgery in Canada. Patterns of endoscope use, attitudes, learning experiences and perceived advantages and challenges regarding endoscopic ear surgery were documented and quantified among Canadian otolaryngologists. Although care should be taken when generalizing these findings to all Canadian otolaryngologists considering the aforementioned study limitations, a number of valuable overall assessments can be offered. Findings show that a considerable number of ear surgeons currently use endoscopes to some capacity in their practice and that, despite some reservations, there is an overall enthusiasm for the endoscopic approach to otologic surgery. Furthermore, there is a general feeling among survey respondents that endoscopes will likely have a role to play in the future of otologic surgery. Given the continuous improvement in endoscopic technology and increasing acceptance of endoscopic ear surgery, investment in training courses and guidance for those looking to start or advance their use of the endoscope in their practice will be vital in the years to come.

## Ethics approval

Ethics approval was obtained from the UBC Behavioural Research Ethics Board (ID H14-03499) and consent was obtained from survey participants by email using the CSO listserv.

## Additional file

Additional file 1:
**Endoscopic ear surgery in Canada survey.** (DOC 25 kb)

## Abbreviations

CSO: Canadian Society of Otolaryngology
OR: Odds ratio
ANOVA: Analysis of variance
