# Supplementary material for: Endoscopic ear surgery in Canada: a cross-sectional study
Source: J Otolaryngol Head Neck Surg. 2016 Jan 19;45:4. doi: 10.1186/s40463-016-0117-7 (PMC4717547; doi:10.1186/s40463-016-0117-7)
Supplement: Additional file 1: — Endoscopic ear surgery in Canada survey. (DOC 25 kb) [file 40463_2016_117_MOESM1_ESM.doc]

**Appendix A**

1. Do you perform ear surgery?
   1. Yes
   2. No
2. How many years have you been practicing otolaryngology?
   1. Please select the number of years (select 0 if you are a trainee)
   2. If you are a trainee, indicate your year of training:
      1. PGY1
      2. PGY2
      3. PGY3
      4. PGY4
      5. PGY5
      6. Fellow
3. How do you use endoscopes in your otology practice?
   1. In the clinic only
   2. In the operating room only
   3. In both the clinic and the operating room
   4. I don’t currently use an endoscope
4. Please select the surgeries for which you are using an endoscope.
   1. Tympanoplasty
   2. Cholesteatoma
   3. Ossicular Chain Reconstruction
   4. Stapedotomy
   5. Skull Base Surgery
   6. None
5. How many endoscopic ear cases have you performed?
   1. 0
   2. 1-10
   3. 11-20
   4. 21-30
   5. 31-50
   6. 50-100
   7. >100
6. Please select the category that most appropriately describes your use of the endscope in cholesteatoma surgery. If you do not use the endoscope in cholesteatoma surgery, please select “Not applicable”.
   1. Using an endoscope at the end of the case to assess for residual disease
   2. Primarily operating with a microscope; use the endoscope sparingly the operate in difficult-to-reach areas
   3. Mainly use the endoscope while reserving the microscope for extensive disease involving the mastoid.
   4. Not applicable.
7. Do you think there is a role for the endoscope in otologic surgery?
   1. Yes
   2. No
   3. Maybe/Unsure
8. Select all below that apply regarding your concerns about the use of an endoscope in ear surgery.
   1. Safety issues
   2. Cost
   3. Technically difficult to use
   4. Efficiency/Operative Time
   5. Other
   6. None
   7. If you selected “Safety Issues” or “Other”, please specify.
9. Select all that apply with regards to the perceived advantages of endoscopic ear surgery.
   1. Safety
   2. Cost
   3. Ease of use
   4. Efficiency/Faster operative time
   5. Reduced recurrence/residual disease rate of cholesteatoma
   6. More likely to preserve ossicular chain
   7. Less post-operative pain
   8. Faster patient recovery
   9. None
10. In comparison to the microscope, how would you describe your overall learning experience with the endoscope in ear surgery?
    1. I find endoscopic ear surgery easier than microscopic surgery
    2. I find endoscopic surgery harder than microscopic surgery
    3. I find endoscopic surgery comparable to microscopic surgery
11. I will likely use endoscopes in the future.
    1. Strongly agree
    2. Agree
    3. Neutral
    4. Disagree
    5. Strongly disagree
